# Controlling Shock-Induced Energy Release Characteristics of PTFE/Al by Adding Oxides

**DOI:** 10.3390/ma15165502

**Published:** 2022-08-10

**Authors:** Ying Yuan, Yiqiang Cai, Dongfang Shi, Pengwan Chen, Rui Liu, Haifu Wang

**Affiliations:** State Key Laboratory of Explosion Science and Technology, Beijing Institute of Technology, Beijing 100811, China

**Keywords:** PTFE/Al/oxide, shock-induced, energy release characteristic, controlling effect, shock wave

## Abstract

Polytetrafluoroethylene (PTFE)/aluminum (Al)-based energetic material is a kind of energetic material with great application potential. In this research, the control of the shock-induced energy release characteristics of PTFE/Al-based energetic material by adding oxides (bismuth trioxide, copper oxide, molybdenum trioxide, and iron trioxide) was studied by experimentation and theoretical analysis. Ballistic impact experiments with impact velocity of 735~1290 m/s showed that the oxides controlled the energy release characteristics by the coupling of impact velocities and oxide characteristics. In these experiments, the overpressure characteristics, including the quasi-static overpressure peak, duration, and impulse, were used to characterize the energy release characteristics. It turned out that when the nominal impact velocity was 735 m/s, the quasi-static overpressure peak of PTFE/Al/MoO_3_ (0.1190 MPa) was 1.99 times higher than that of PTFE/Al (0.0598 MPa). Based on these experimental results, an analytical model was developed indicating that the apparent activation energy and impact shock pressure dominated the energy release characteristic of PTFE/Al/oxide. This controlling mechanism indicated that oxides enhanced the reaction after shock wave unloading, and the chemical and physical properties of the corresponding thermites also affected the energy release characteristics. These conclusions can guide the design of PTFE-based energetic materials, especially the application of oxides in PTFE-based reactive materials.

## 1. Introduction

Polytetrafluoroethylene (PTFE)/Aluminum (Al), as a novel energetic material, is extensively utilized in explosion and warhead terminal damage due to its unique impact reaction characteristics and high energy density (21 kJ/cm^3^) [1,2].

In recent years, a lot of studies have been conducted on the chemical reaction of PTFE/Al. The noticeable decomposition of PTFE/Al occurs at temperature above 673 K, and the main reaction products involve AlF_3_, CO, and CO_2_ [3]. A standardized evaluation technique for characterizing the energy release of PTFE/Al, which offers a feasible pathway to present the energy release of PTFE/Al quantitatively, has been developed [4,5,6]. Furthermore, considering the energy consumption of the test chamber and the energy of leakage gas, a more perfect method for calculating and measuring the impact energy release of active materials has been developed [7].

However, its applications are restricted by its low mechanical strength and low reaction efficiency due to non-self-sustaining reactions. Many energetic components, such as hydrides [8,9,10,11], active metals [12,13,14], and oxides, have been introduced to PTFE/Al to enhance its energy release characteristics. Among them, the effects of adding oxides on the energy release characteristics of PTFE/Al has received much attention from scholars due to the excellent reaction performance and various reaction characteristics of thermite (Al/oxide). Experiments have been conducted by self-designed energy release testing devices and the results have shown that CuO promotes the energy release efficiency of PTFE/Al [15]. Drop-weight tests have been conducted, indicating that Bi_2_O_3_ improves the impact sensitivity of PTFE/Al [16]. In addition, the burning speed, specific volume, and mechanical properties of PTFE/Al/Fe_2_O_3_ [17], and the mechanical and reaction properties and thermal decomposition of PTFE/Al/MnO_2_ [18] have also been studied.

These pioneering works indeed have demonstrated the potential of oxides in adjusting the energy release characteristics of PTFE/Al energetic material. However, the lack of a systematic study on how the oxides control the energy release characteristics of PTFE/Al-based energetic material seriously restricts further application of PTFE/Al/oxide in weapons.

In this work, PTFE/Al and four kinds of PTFE/Al/oxide, including bismuth trioxide (Bi_2_O_3_), copper oxide (CuO), molybdenum trioxide (MoO_3_), and iron trioxide (Fe_2_O_3_), were fabricated to investigate the shock-induced energy release characteristics by vented-chamber tests. An analytical model was developed to discuss how the oxides control the shock-induced energy release characteristics of PTFE/Al-based energetic material. The results revealed the mechanism of oxides controlling shock-induced reactions and can guide the design and application of reactive materials. In the Section 1, the development of PTFE-based reactive materials was introduced, and the studies on PTFE/Al/oxide were summarized. Imperfections in published studies were pointed out. In the Section 2, the sample preparation and energy release test setup is introduced in detail. In the Section 3, the shock-induced energy release behavior of the samples is introduced, and an analytical model is established to quantitatively describe the shock-induced energy release of reactive materials. Combined with the analytical model, the energy release characteristics of different types of reactive materials were analyzed when they impacted with 735~1290 m/s. In the Section 4, the results, analysis, and discussions are concluded.

## 2. Materials and Methods

### 2.1. Sample Preparation

There were five kinds of energetic materials fabricated in this work. The raw powders were: Al (2.78 g/cm^3^, FLQT2, from Xingrongyuan, Beijing, China), PTFE (2.20 g/cm^3^, MP1300, from dongfu, Shanghai, China), Bi_2_O_3_ (8.90 g/cm^3^, 325 mesh, from Xingrongyuan, Beijing, China), CuO (6.50 g/cm^3^, 325 mesh, from Xingrongyuan, Beijing, China), Fe_2_O_3_ (5.24 g/cm^3^, 325 mesh, from Xingrongyuan, Beijing, China), and MoO_3_ (4.69 g/cm^3^, 325 mesh, from Xingrongyuan, Beijing, China). The chemical reaction information of the involved energetic mixtures is listed in Table 1.

According to the stoichiometric ratio of each reaction, the PTFE/Al-based energetic materials were mixed with 20 wt.% oxide to meet the oxygen equilibrium. The specific information of samples is listed in Table 2. The actual density in Table 2 was calculated according to the actual size and mass of the sample after preparation, in which PTFE/Al/Fe_2_O_3_ had the lowest relative density and PTFE/Al/Bi_2_O_3_ had the highest relative density.

The preparation process mainly included mixing, cold isostatic pressing, and high-temperature sintering. Firstly, the raw powders of a certain mass were added to the anhydrous ethanol solution and mixed by a blender for about 60 min, followed by a drying process at room temperature lasting 48 h. Then, the mixed powder was filled into a mold with an inner diameter of 10 mm and uniaxially cold-pressed at about 250 MPa. Finally, the cold isostatic pressing samples were placed in a vacuum sintering oven. The oven temperature rose to 370 °C at a rate of 60 °C/h, then held at 370 °C for 4.5 h, and finally brought down to room temperature at a rate of 60 °C/h.

**Figure 1:**
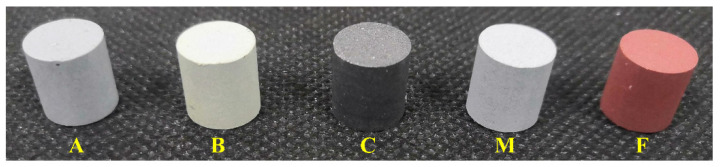
Morphology of PTFE/Al/oxides: A, B, C, M, and F are PTFE/Al, PTFE/Al/Bi_2_O_3_, PTFE/Al/ CuO, PTFE/Al/ MoO_3_, and PTFE/Al/ Fe_2_O_3_, respectively.

**Figure 2:**
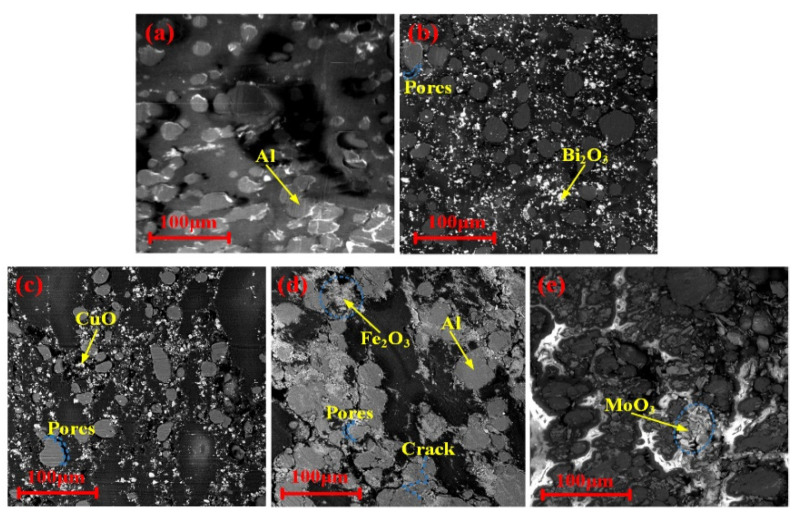
Microscopic characteristics of PTFE/Al/oxides: (**a**) PTFE/Al; (**b**) PTFE/Al/Bi_2_O_3_; (**c**) PTFE/Al/CuO; (**d**) PTFE/Al/MoO_3_; (**e**) PTFE/Al/Fe_2_O_3_.

The typical prepared PTFE/Al/oxide samples with Φ10 mm × 10 mm are shown in Figure 1. As shown in Figure 1, the PTFE/Al-based energetic materials differed in color, presenting gray-green (Bi_2_O_3_), black (CuO), gray-white (MoO_3_), and red (Fe_2_O_3_). The microstructure of the PTFE/Al/oxides is shown in Figure 2. Al particles and oxide particles were wrapped in PTFE matrix, and there were a few pores between the matrix and particles and in the matrix itself. Among them, the PTFE/Al, PTFE/Al/Bi_2_O_3,_ and PTFE/Al/CuO particles were evenly distributed and closely bonded with the matrix. The MoO_3_ in the PTFE/Al/MoO_3_ had an agglomeration phenomenon, and the Fe_2_O_3_ showed obvious porous characteristics in the PTFE/Al/Fe_2_O_3_.

### 2.2. Experimental Setup

**Figure 3:**
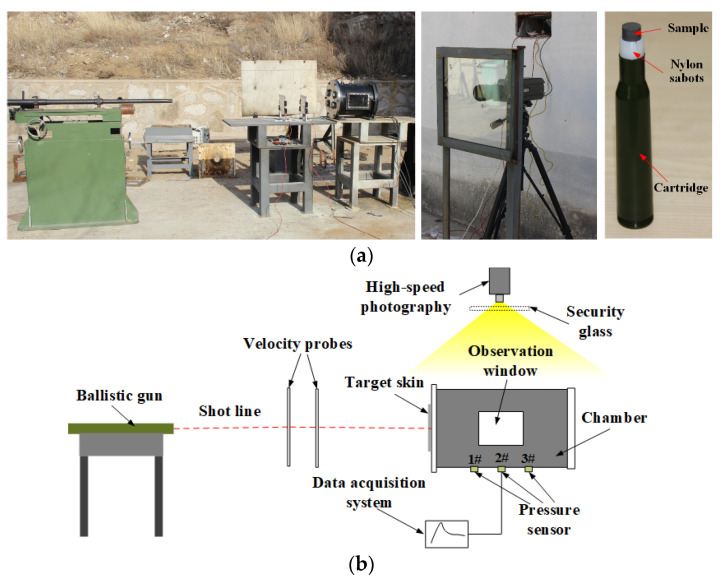
Experimental setup: (**a**) physical; (**b**) schematic.

Figure 3 presents the experimental setup used to investigate the shock-induced energy release characteristics by the quasi-vented-chamber calorimetry technique. The test system mainly included a ballistic gun, chamber, pressure sensors (AK-1, measuring range from 0 to 1 MPa, sampling frequency 1 MHz), data acquisition system (TST3206), and velocity measuring instrumentation. The samples, encapsulated in nylon sabots, as shown in Figure 3a, were launched from the ballistic gun with a diameter of 12.7 mm. The velocity of the sample was controlled by adjusting the mass of gunpowder loaded into the cartridge. However, the combustion of gunpowder is complicated and affected by many factors, so the projectile velocity fluctuated within a certain range when the same charge was filled.

The chamber with a volume of 27.35 L was sealed initially with a thin-skin plate (2024-T3 aluminum, thicknesses of 3 mm) at one end. In the interior of the chamber there was a hardened steel anvil on the other end for the energetic material to impact after passing through the target skin. In the experiment, it was considered that the test tank was a rigid body, and that no deformation occurred during the reaction of the energetic material. Three sensors were arranged in an equidistant sequence parallel with the axis of the chamber to record the overpressure characteristics. The pressure sensor was close to the inner wall of the chamber, and the sensor data was transmitted to the data processing system through signal lines. When the pressure in the chamber increased, the sensor acted as a pressure-sensitive material, and its resistance changed with the pressure change to correspond to the electrical signal change and recorded the pressure change in the chamber. The sensor began to record the experimental data when the pressure in the tank exceeded 3% of the medium range. The experiment under the same conditions was carried out three times to exclude accidental errors.

The Phantom V710 high-speed photography camera (Vision Research, Inc., Wayne, NJ, USA) was used to record the shock-induced energy release characteristics of the PTFE/Al/oxide materials. The selected frame rate was 20,000 fps so that a frame was taken every 50 μs. The resolution was 640 × 480 pixels and the exposure time was set to 10 μs. These settings were selected based on early testing and represent an optimal tradeoff between available lighting and the minimization of blur in the images.

## 3. Results and Discussion

### 3.1. Typical Shock-Induced Energy Release Characteristics

**Figure 4:**
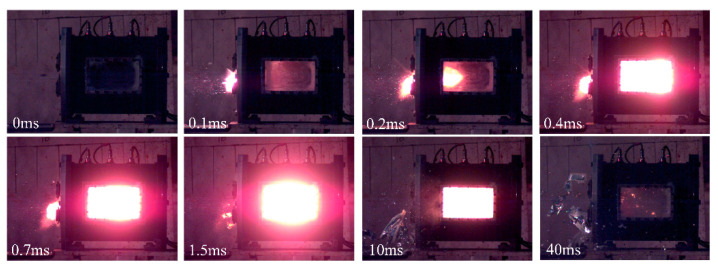
Typical shock-induced reaction phenomena (PTFE/Al/MoO_3_, 910.45 m/s).

The energetic materials launched by the ballistic gun perforated the target skin of the chamber, and entered the test chamber with a violent reaction. The typical shock-induced reaction phenomena of the PTFE/Al/oxide energetic materials are shown in Figure 4. As shown in Figure 4, when the energetic materials impacted the skin plate, some debris of the energetic materials were formed and reacted outside the chamber (as shown in sequence 0.1 ms). It can be observed that energetic material started to react to an extent and continued on to the impact anvil (as shown in sequence 0.2 ms). When the energetic material impacted the anvil inside the test chamber, the energetic material had a more violent reaction (as shown in sequence 0.4 ms). The reaction in the test chamber lasted for tens of microseconds and then stopped gradually.

**Figure 5:**
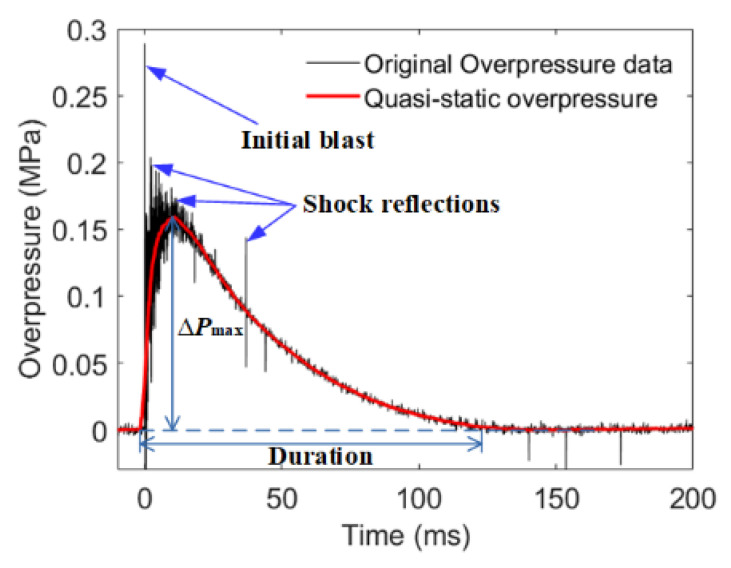
The typical overpressure in the chamber varied with time.

The violent exothermic reaction triggered overpressure in the test chamber, and the typical variation in overpressure with time is shown in Figure 5. As shown in Figure 5, the overpressure firstly went through a very high peak, followed by a relatively high quasi-static overpressure peak (∆*P*_max_). The first extremely high peak was caused by the initial blast of the energetic material, and the subsequent quasi-static overpressure peak was caused by the heat release by the energetic material reaction. After that, the high-pressure gas in the chamber leaked out, and the pressure in the chamber decreased. An analysis that combines the overpressure variation with high-speed photographic frames indicates that the peak of overpressure lagged behind, in time scale, the most intense reaction of energetic materials. This strongly suggests that the quasi-static overpressure in the test chamber characterized the accumulated energy released by the energetic material in the test chamber, not the reaction intensity instantaneously. Because the blast peak pressure had the characteristics of short-time and rapid attenuation, the measured peak pressure was greatly affected by the sampling frequency and sensor position. So, quasi-static overpressure was used to characterize the energy release of the shock-induced reaction.

The energy released by the energetic material was adopted to characterize the quasi-static overpressure peak (Δ*P*_max_). Ignoring the influence of the reaction products in the gas, which is very little, and the gas leakage through perforation, which is negligible during such a short time, it was assumed that the heat released by the energetic material is all used to heat the initial gas in the chamber. The relationship between the quasi-static overpressure peak in the chamber and the energy released by the energetic material in the chamber can be expressed as [7]

(1) $$\Delta P_{\max}=\frac{\gamma_{a}-1}{V}\Delta E,$$

where Δ*E* is the released energy, *V* is the volume of the test chamber and *γ_a_* is the ratio of the specific heat of the gas. The reaction efficiency of the PTFE/Al/oxide can be expressed as

(2) $$\eta =\frac{\Delta E}{\Delta E_{t}},$$

where *η* is the reaction efficiency of energetic materials, and Δ*E_t_* is the theoretical total energy of the sample, which ignores the heat released by further side reaction.

### 3.2. Analytical Model of Shock-Induced Energy Release Characteristics

The shock-induced energy release mechanism of PTFE/Al/oxide is complex due to the combination effect of mechanics-thematic chemistry. When PTFE/Al/oxide energetic material impacts the skin plate, the shock wave is generated and propagates within the energetic material. Upon the adiabatic compression of the shock wave, the temperature of the energetic material increases, triggering the chemical reaction of the PTFE/Al/oxide. Based on the one-dimensional shock wave theory and the conservation of mass and momentum at the impact interface, the initial shock wave induced by the impact within the PTFE/Al/oxide can be expressed as

(3) $$P_{0}=v_{0}\frac{\rho_{p0}\rho_{t0}U_{p}U_{t}}{\rho_{p0}U_{p}+\rho_{t0}U_{t}},$$

where *P*_0_ is the initial impact shock pressure, *v*_0_ is the impact velocity of the energetic projectile, *U* is the shock wave velocity, and *ρ*_0_ is the initial density. Subscripts *p* and *t* represent energetic projectile and plate, respectively. The relationship between the shock velocity and particle velocity can be expressed as

(4) $$\{\begin{array}{c} U_{p}=C_{p}+S_{p}u_{p}\\ U_{t}=C_{t}+S_{t}u_{t} \end{array},$$

where *C* is the sound speed of the material, *S_p_* is the Hugoniot parameter, and *u* is the particle velocity. The temperature of the energetic material rises under the compression of the shock wave, which induces a chemical reaction. The relationship between the shock wave pressure and temperature can be expressed as [20]

(5) $$\begin{array}{l} T=T_{0}\exp\left[\left(\frac{\gamma_{0}}{V_{0}}\right)\left(V_{0}-V_{1}\right)\right]+\frac{V_{0}-V_{1}}{2C_{V}}P\\ +\frac{\exp\left[\left(-\gamma_{0}/V_{0}\right)V_{1}\right]}{2C_{V}}\int_{V_{0}}^{V_{1}}P\exp\left(\frac{\gamma_{0}}{V_{0}}V\right)\left[2-\frac{\gamma_{0}}{V_{0}}\left(V_{0}-V\right)\right]dV \end{array}$$

where *T* is the temperature, *γ* is the Gruneisen coefficient, *V* is the specific volume, *C_V_* is the heat capacity at constant volume, and *P* is the shock pressure. Subscript 0 represents the material parameters in the initial state, and subscript 1 represents the parameters after the shock wave compression state.

It is assumed that the PTFE/Al/oxide chemical reaction efficiency varies linearly with time. According to the research of Ortega [21], the relationship between the reaction efficiency *η* and temperature *T* of the energetic material can be expressed as

(6) $$\frac{dT}{d\eta }=\frac{R_{u}T^{2}}{E_{a}}\left[\frac{1}{2\eta }-\frac{n\ln\left(1-\eta \right)+n-1}{n\left(1-\eta \right)\left[-\ln\left(1-\eta \right)\right]}\right],$$

where *R*_u_ is the universal gas constant, which is 8.314 J/(mol K), *E_a_* is the apparent activation energy, and *n* is the chemical coefficient related to boundary conditions and reaction mechanisms. As a typical composite energetic material, the material parameters of PTFE/Al/oxide can be estimated by its composition and content as follows [22]

(7) $$\chi =\sum_{i=1}^{n}\chi_{i}m_{i},$$

where *χ* is the material parameter, such as the sound velocity *C*, Hugoniot parameter *S*, heat capacity *C_V_*, Gruneisen coefficient *γ*, chemical coefficient related to boundary conditions and reaction mechanisms *n*, and the apparent activation energy *E_a_*, *χ_i_* is the material parameter of each specific composition, and *m_i_* is the mass ratio of each specific composition.

The reaction parameters of PTFE/Al/oxide, such as the chemical reaction coefficient and apparent activation energy, are approximated by considering the two kinds of reaction parameters. The chemical reaction coefficient of PTFE/Al and Al/oxide are 0.625 [23] and 0.1 [24], respectively. The apparent activation energy of PTFE/Al is 50.836 kJ mol^−1^ [23]. The activation energy of Al/oxide can be calculated by the Arrhenius kinetic model approach of the Flynn–Wall–Ozawa isoconversion method [25]. Some materials and reaction parameters involved in the calculation of the model are listed in Table 3. The effects of some properties of oxides (*S*, *C*, and *γ*) on the PTFE/Al/oxide properties are not considered temporarily and are replaced by those of Bi_2_O_3_ [26].

### 3.3. Overpressure Characteristics

**Figure 6:**
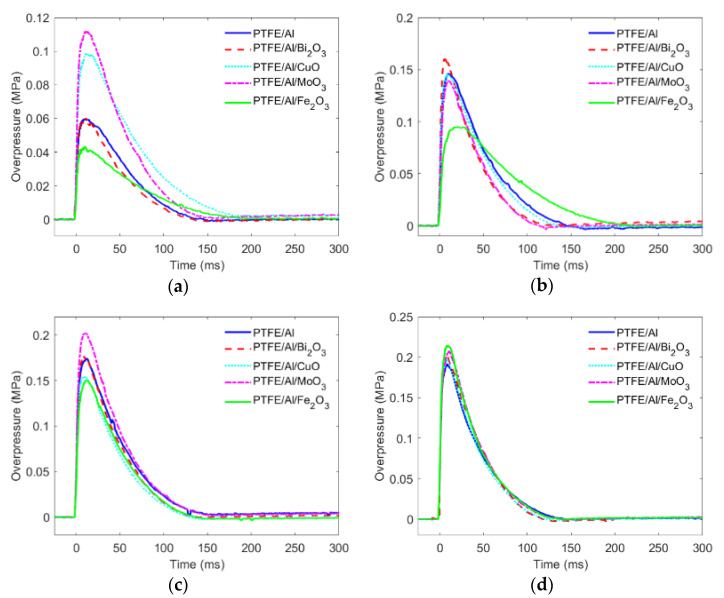
The quasi-static overpressure varying time: (**a**) nominal velocity 735 m/s; (**b**) nominal velocity 920 m/s; (**c**) nominal velocity 1127 m/s; (**d**) nominal velocity 1290 m/s.

Figure 6 presents the quasi-static pressure vs. time induced by the PTFE/Al/oxide with impacting at different velocities. The quasi-static overpressure characteristics of the PTFE/Al/oxide with different impact velocities are listed in Table 4. The experimental results indicate that the controlling effect of oxides on overpressures depended on the oxide type and impact velocity.

As shown in Figure 6 and Table 4, the impact velocity was the most primary factor affecting the overpressure characteristics. In general, the quasi-static overpressure peak increased monotonically with the increase in impact velocity. For PTFE/Al, with the impact velocity increasing for 726.8 m/s to 1345.8 m/s, Δ*P*_max_ increased from 0.0598 MPa to 0.1917 MPa. Taking PTFE/Al/Fe_2_O_3_ for example, which was most affected by the velocity in the PTFE/Al/oxide reactive materials, the overpressure increased from 0.0434 MPa to 0.2150 MPa as the velocity increased from 741.07 m/s to 1299.46 m/s. This is because the intensity of the shock wave in the energetic material increased and the energy release efficiency of the energetic material increased. The variation trend of the overpressure duration was not obvious, and the variation rule of each type of energetic material was different, ranging from 100 ms and 200 ms. With the increase in impact velocity, the impulse generally increased. Impulse, a parameter that comprehensively considers the overpressure intensity and duration, can more reasonably characterize the performance of the energy release of energetic materials.

Obviously, different types of PTFE/Al/oxide showed different energy release characteristics under different impact velocities. When the nominal velocity was 735 m/s, the quasi-static overpressure induced by MoO_3_ was the highest, and the impulse effect induced by copper oxide was the strongest. When the nominal velocity was 920 m/s, the quasi-static overpressure induced by Fe_2_O_3_ was significantly lower than that of the other four kinds of PTFE/Al/oxide energetic materials, with little difference in impulse. When the nominal velocity was 1127 m/s, the quasi-static overpressure peak and impulse induced by molybdenum oxide were the highest. When the nominal velocity was 1290 m/s, the quasi-static overpressure peak and impulse of the five kinds of energetic materials were basically the same.

### 3.4. Energy Release Efficiency of PTFE/Al/Oxide

**Figure 7:**
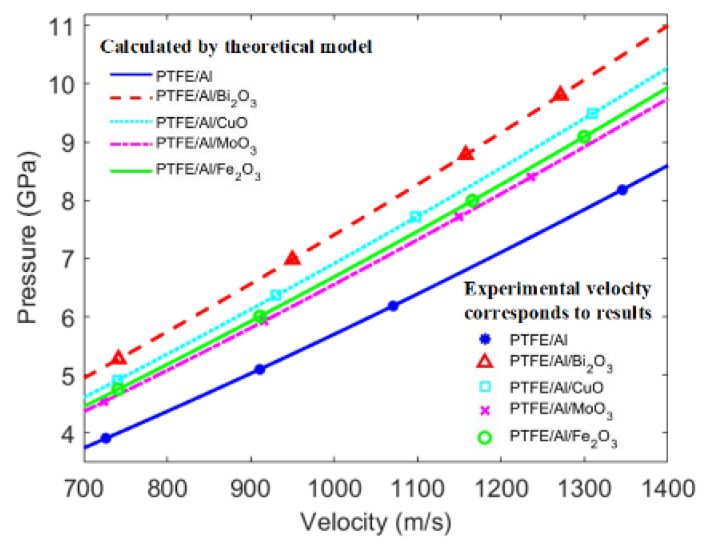
Pressure varying with impact velocity.

Based on the shock-induced reaction model of PTFE/Al/oxide, the initial impact shock pressures of PTFE/Al/oxide with different impact velocities are presented in Figure 7. As shown in Figure 7, under the same impact velocity, the initial pressure of all kinds of PTFE/Al/oxide were higher than that of PTFE/Al, which can be attributed to the high shock impedance of the oxide. Among them, under the same nominal velocity, the initial impact pressure within PTFE/Al/Bi_2_O_3_ was highest. This is because Bi_2_O_3_ has the highest density, which is conducive to promoting the impact impedance of energetic material. In addition, with the increase in impact velocity, the increase extent of pressure of PTFE/Al/oxide increased compared with that of PTFE/Al. The analysis indicates that the high-density oxides controlled the shock-induced energy release characteristics of PTFE/Al-based energetic materials by increasing the initial impact pressure.

**Figure 8:**
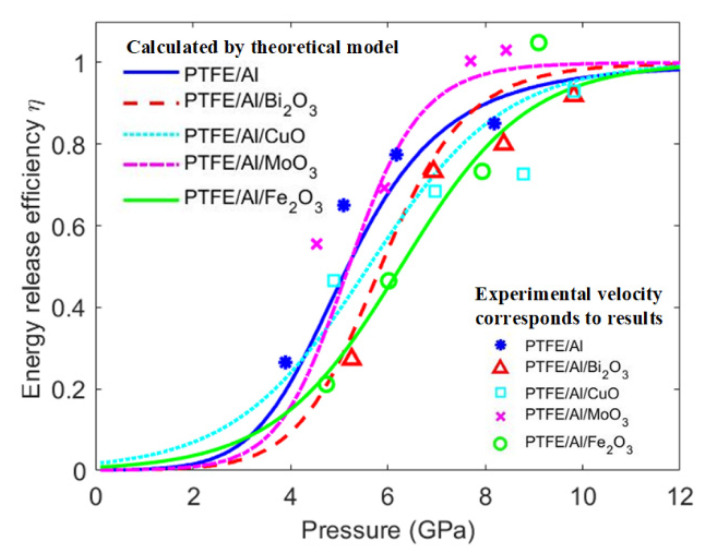
Energy release efficiency of PTFE/Al/oxide varying with impact pressure.

Based on the analytical model, considering the influence of the chemical reaction characteristics and apparent activation energy of energetic materials with different oxides, the energetic release efficiency of the PTFE/Al/oxide varying with impact pressure is shown in Figure 8. As shown in Figure 8, in terms of the analytical model predictions, with the increase in impact pressure, the energetic release efficiency of the PTFE/Al/oxide increased in an S-shaped tendency [28]. When the impact pressure was low (<2 GPa) or high (>10 GPa) enough, the energetic release efficiency increased slowly with impact pressure increasing. However, there are some differences in the specific change law of PTFE/Al/oxide, mainly due to the difference in activation energy and reaction coefficient of the different PTFE/Al/oxide energetic materials. On the whole, with impact pressure increasing, the energy release efficiency of PTFE/Al with MoO_3_ increased at the fastest rate, and the energetic release efficiency of PTFE/Al with Fe_2_O_3_ increased at the slowest rate.

In the above analysis, it was assumed that all the energetic materials reacted in the test chamber without considering the mass of backsplash debris, and the attenuation of the shock wave during the propagation was not considered. Such an assumption will result in the energy release efficiency by calculation being higher than the actual energy release efficiency but will not affect the relative law. The shock-induced energy release characteristics have also been studied by experiments in recent research [29]. The predictions of the analytical model are also in good agreement with the literature [29].

### 3.5. Controlling Mechanism of Oxides on Energy Release Characteristics

In this section, the comprehensive effect of oxides on the energy release characteristics of PTFE/Al energetic materials is discussed based on the reaction mechanism. The response behaviors of PTFE/Al-based energetic materials can be distinguished as four classes, reacting from weak to strong. For Type I, no chemical reaction occurs, and the energetic materials only become densified and homogenized; for Type II, partial chemical reaction occurs in the energetic material, and the reaction stops when the pressure decays; for Type III, chemical reaction occurs in the energetic material, and the reaction continues as a self-sustaining chemical reaction after pressure unloading; for Type IV, complete chemical reaction occurs.

Under the experimental conditions in this study, as the impact velocity increased, the shock-induced reaction of energetic materials underwent Type II, Type III, and Type IV, gradually. This means that, when impact velocity was high, the energetic materials reacted completely (Type IV). In this case, the energy released by energetic materials was mainly determined by the total energy content. The energy released by the energetic materials was determined by the mass and energy release efficiency of the energetic materials involved in the reaction at other impact loads.

The control effect of the oxides on the shock-induced energy release of the energetic materials mainly reflected in controlling the Type II or Type III energetic material response. Thermites showed more significant self-sustaining property than Al/PTFE, and the addition of oxides made the reaction type of energetic materials evolve from Type II to Type III, which improvex the energy release efficiency of energetic materials at a relatively low velocity. It is worth noting that the addition of different oxides also lead to some differences in the controlling effect, which was determined by the specific properties of the various thermites.

When the impact velocity ranged from 723.98 m/s to 1345.80 m/s, MoO_3_ presented the best optimization enhancement effect on the reaction performance of PTFE/Al because of the comprehensive effect of the highest heat of Al/MoO_3_ per unit mass (4.698 kJ/g), the lower ignition temperature of Al/MoO_3_ (~880 K) [26], and the further reaction between reaction products (MoC_2_).

Note that the control effect of Fe_2_O_3_ strongly depended on the impact velocity. It was attributed that as Al/Fe_2_O_3_ has a high onset reaction temperature (~937 K) [26], it was difficult to start its reaction under low-velocity impact, leading to the decrease in the overall energy release efficiency of the energetic material. The other reason is that the energy release per unit mass of Al/Fe_2_O_3_ is lower than that of Al/PTFE, and the additional Fe_2_O_3_ reduced the total energy content of energetic material.

The addition of Bi_2_O_3_ in PTFE/Al improved ∆*P*_max_ of the energetic materials modestly. Since the reaction temperature of the energetic materials exceeded 3000 K, the reaction product Bi formed vapor, which increased the amount of gas produced in the reaction of the energetic materials and raised the overpressure. However, the increase in gaseous product volume had a limited contribution to the increase in overpressure due to the large volume of the test chamber used in the experiments. It can be inferred that if PTFE/Al/Bi_2_O_3_ were to react in a relatively narrow space, its overpressure peak would be higher than that of other PTFE/Al/oxide energetic materials.

The analyses above indicate that the overpressure of the PTFE/Al/oxide was controlled by many factors, such as the specific heat capacity of the oxide, the reaction onset temperature of the thermite, the gas product volume of reaction, and so on.

## 4. Conclusions

The shock-induced energy release characteristics of PTFE/Al-based energetic material with oxides (Bi_2_O_3_, CuO, MoO_3_, and Fe_2_O_3_) were studied by vented-chamber tests and by theoretical analysis. The overpressure characteristics were analyzed with consideration of the shock wave and activation energy. Furthermore, the controlling effect of oxides on PTFE/Al shock-induced energy release characteristics was analyzed and discussed. The main conclusions are drawn as follows:

(a) The experimental results indicate that the oxides controlled the shock-induced energy release characteristics, and this controlling effect was affected by the impact velocity. With a lower impact velocity (usually lower than 750 m/s), the energy release characteristics of the PTFE/Al was significantly enhanced by MoO_3_, by 1.99 times. The oxides also presented a significant influence on the overpressure duration of the PTFE/Al-based energetic materials.
(b) The analytical model for PTFE/Al/oxide shock-induced energy release indicated that the oxides dominated the energy release characteristics by affecting the apparent activation energy and impact shock pressure of the energetic materials. Oxides with a high-sensitivity corresponding thermite, or with a high density could enhance the energy release performance of PTFE/Al/oxide.
(c) The mechanism of oxides controlling the shock-induced energetic behaviors of PTFE/Al energetic materials was revealed. It indicated that oxides improved the continuous reaction ability of energetic materials after shock wave unloading. The controlling effects of different oxides was determined by the chemical and physical properties of the corresponding thermites.
(d) This study fills the gap in the theoretical study of PTFE/Al shock-induced energy release behaviors and has great guiding significance for the design and application of energetic materials.

## Figures and Tables

**Table 1:** Summary of chemical reaction information for mixtures [19].

Mixture	Chemical Reaction Equation	Stoichiometric Ratio	Theoretical Δ*H* (J/g)
Al/PTFE	4Al + 3C_2_F_4_→4AlF_3_ + 6C	26.5/73.5	8530
Al/Bi_2_O_3_	2Al + Bi_2_O_3_→Al_2_O_3_ + 3Bi	10.4/89.6	2115
Al/CuO	2Al + 3CuO→Al_2_O_3_ + 3Cu	18.4/81.6	4072
Al/MoO_3_	2Al + MoO_3_→Al_2_O_3_ + Mo	27.3/72.7	4698
Al/Fe_2_O_3_	2Al + Fe_2_O_3_→Al_2_O_3_ + 2Fe	25.3/74.7	3156

**Table 2:** Specific information of PTFE/Al/oxide.

Type	Oxide	PTFE/Al/Oxide ^a^	*ρ*_TMD_/*ρ*_a_^b^ (g/cm^3^)	Relative Density	*E_t_* ^c^ (kJ/g)
A	/	73.5/26.5	2.33/2.28	97.6%	8.528
B	Bi_2_O_3_	57.1/22.9/20.0	2.74/2.73	99.6%	7.098
C	CuO	55.5/24.5/20.0	2.69/2.64	98.1%	7.418
M	MoO_3_	53.3/26.7/20.0	2.63/2.53	96.2%	7.467
F	Fe_2_O_3_	53.8/26.2/20.0	2.65/2.48	93.6%	7.586

^a^ The mass fraction; ^b^ theoretical maximum density/actual density; ^c^ theoretical total energy.

**Table 3:** Material parameter of oxide [27].

Oxide Type	Bi_2_O_3_	CuO	Fe_2_O_3_	MoO_3_
*C_v_* (J/(mol K))	236	530	662	521
*E_a_* ^1^ (kJ/mol)	201.5	349.5	425.4	252.3

^1^ represents the apparent activation energy of the corresponding thermite.

**Table 4:** The quasi-static overpressure characteristic.

Sample	Impact Velocity (m/s)	Δ*P*_max_ (MPa)	Duration (ms)	Impulse (s kPa)
A	726.80	0.0598	126.76	3.6795
B	741.48	0.0602	118.88	3.2596
C	739.97	0.0987	182.44	7.1655
M	723.98	0.1190	138.96	6.5227
F	741.07	0.0434	166.72	3.1393
A	910.54	0.1466	139.74	8.2577
B	949.80	0.1619	117.04	6.7877
C	930.28	0.1456	126.40	7.6622
M	915.29	0.1397	106.38	6.4186
F	911.87	0.0953	198.96	8.5054
A	1070.43	0.1745	127.42	9.5816
B	1157.47	0.1767	125.70	8.9589
C	1098.18	0.1544	123.02	7.7877
M	1149.03	0.2023	152.02	10.883
F	1165.57	0.1503	129.08	8.1513
A	1345.80	0.1917	131.30	9.1400
B	1270.97	0.2029	114.77	9.3705
C	1309.50	0.1978	115.84	8.7886
M	1235.94	0.2078	127.32	9.7313
F	1299.46	0.2150	124.12	9.8345

## Data Availability

Not applicable.
